# Clinical characteristics and cytokine profiles of children with acute lower respiratory tract infections caused by human rhinovirus

**DOI:** 10.1371/journal.pone.0198624

**Published:** 2018-07-03

**Authors:** Jong Gyun Ahn, Dong Soo Kim, Ki Hwan Kim

**Affiliations:** 1 Department of Pediatrics, Severance Children’s Hospital, Yonsei University College of Medicine, Seoul, Republic of Korea; 2 Department of Medicine, The Graduate School of Yonsei University, Seoul, Republic of Korea; Telethon Institute for Child Health Research, AUSTRALIA

## Abstract

The clinical profile of human rhinovirus (HRV) with regard to lower respiratory infections remains unclear. We analyzed the clinical features and cytokine responses of HRV isolates in children with respiratory infections. Quantitative analysis and genotyping of the HRV-positive samples from 601 nasopharyngeal aspirates (NPAs) were performed using VP4/VP2 sequencing. To compare T-helper1 (Th1) type (IFN-γ, TNF-α) and Th2 type (IL-4, IL-10) cytokine responses between HRV-A, B and C, the levels of the four cytokines were measured. The HRV-positive children had shorter fever duration (*P* = 0.018), and higher frequencies of chest retraction (*P* = 0.002) and wheezing (*P* = 0.022) than did the HRV-negative group. HRV-A was identified in 55 cases (58.5%), HRV-B in 8 (8.5%), and HRV-C in 31 (33.0%). There were no significant differences in the clinical data or NPA cytokines levels between patients with HRV-A and HRV-C infections. HRV is an important pathogen of the lower respiratory tract in young children. HRV-A and HRV-C are the dominant species that cause respiratory difficulty in young children.

## Introduction

Human rhinovirus (HRV) is the most common viral respiratory agent in humans. Although it is the predominant cause of the common cold, HRV was recently found to be associated with an extensive range of more severe respiratory illnesses. This virus has been implicated in pneumonia, bronchiolitis, and exacerbation of asthma and chronic obstructive pulmonary disease [1–4].

HRV is a small, non-enveloped single-stranded RNA virus that belongs to the *Enterovirus* genus and *Picornaviridae* family. It is currently classified into three species on the basis of gene sequencing analysis. These include HRV-A, HRV-B, and the recently discovered HRV-C [5,6]. There are >100 distinct serotypes of HRV species according to their surface proteins. This biological diversity makes it difficult to develop vaccines or antivirals against HRV. Understanding the epidemiological, clinical, and immunological features of HRV will help to prevent and treat HRV respiratory diseases. However, these characteristics have yet to be completely assessed. Therefore, in this study, we investigated the epidemiological, clinical, and virological characteristics of HRV infections in children with acute lower respiratory tract infections. We also compared the T-helper1(Th1)- and Th2-related cytokine profiles of HRV infection in nasopharyngeal aspirates (NPAs) to assess the immune responses of the disease.

## Materials and methods

### Study patients and sample collection

A total of 601 nasopharyngeal aspirates (NPAs) were collected from children <18 years old hospitalized with acute lower respiratory tract infections at Severance Children`s Hospital in Seoul, Korea between March 2011 and January 2012. As part of the diagnosis, pneumonia, croup, bronchiolitis, and asthma were included. These diseases were diagnosed through physical examination and chest X-rays. Patients were excluded if they were immunocompromised due to HIV infection, malignancy, congenital or acquired immunodeficiency, solid organ transplantation or use of immunosuppressive drugs such as long-term systemic corticosteroids or radiation therapy. NPAs were obtained by suction using a fine, flexible plastic catheter and syringe. After collection, NPAs were immediately transported at 4°C to the laboratory, where they were stored at -70°C until use. Patient demographic and clinical data including medical history, detailed signs and symptoms, physical examination, and laboratory and radiology results were collected from a retrospective review of the medical records and interviews upon admission. Disease severity was assessed according to a respiratory symptom scoring system based on previously published studies [7,8].

### Multiplex PCR/RT-PCR for HRV and other respiratory viruses

Nucleic acid from each NPA was extracted using a QIAamp Viral Mini kit (QIAGEN, Hilden, Germany) according to the manufacturer`s instructions. A one-step multiplex PCR/RT-PCR kit (SolGent, Daejeon, Korea) was used to detect 12 respiratory viruses [9]. These included HRV, RSV, human bocavirus (HBoV), influenza A virus (IAV), influenza B virus (IBV), human metapneumovirus (HMPV), adenovirus (ADV), human coronaviruses (HCoV) 229E, OC43, and parainfluenza viruses (PIV) 1–3. Amplification was performed according to the manufacturer`s protocol. The PCR products were run on a 2% agarose gel, stained with ethidium bromide, and visualized with UV light.

### Real-time RT-PCR and sequence analysis of HRV

HRV-positive specimens by multiplex PCR/RT-PCR were subsequently submitted to amplification reaction with the real-time RT-PCR assay using the PowerChek™ Rhinovirus Real-time PCR Kit (Kogenebiotech, Seoul, Korea) and ABI-7500 fast Real-Time PCR System (Applied Biosystems, Foster City, CA). RT-PCR was performed according to the manufacturer’s instructions under the following conditions: initial denaturation at 50°C for 30 min and 95°C for 15 min, followed by 40 cycles at 95°C for 15 s and 60°C for 1 min.

Ninety-four of the 98 HRV-Positive PCR samples were sequenced by SolGent Co. (Daejeon, Korea). The VP4/VP2 sequences were aligned by CLUSTRAL W. Phylogenetic analysis was conducted using MEGA 5.0 software. The nucleotide and deduced amino acid sequences of the VP4/VP2 regions were compared with reference sequences of HRV-A, HRV-B and HRV-C strains previously published in the GenBank using BLAST (Basic Local Alignment Search Tool –http://blast.ncbi.nlm.nih.gov/).

### Cytokine analysis

We measured the concentration of the Th1 (IFN-γ, TNF-α) and Th2 (IL-4, IL-10) cytokines in NPA from 94 HRV-positive patients for which genotyping using VP4/VP2 sequencing was undertaken. The concentrations of the four cytokines in NPA was measured using commercially available enzyme-linked immunosorbent assay kits (eBioscience, San Diego, CA, USA) according to the manufacturer’s instructions.

### Statistical analysis

Statistical analysis was performed using SPSS (version 18.0, Chicago, IL, USA). Demographic, clinical, and laboratory parameters were compared between HRV-positive and -negative children and also among HRV species. Pearson χ^2^ or Fisher’s exact test was applied for categorical variables. The student’s t-test or non-parametric Mann-Whitney U test, ANOVA tests or Kruskal–Wallis test were used for continuous variables, where applicable. *P* values <0.05 were considered statistically significant.

### Ethical approval

The study protocol was reviewed and approved by the Yonsei University Health System Institutional Review Board, Seoul, Korea (4-2008-0649). The study was conducted in accordance with good clinical practices (national regulations and ICH E6) and the principles of the Helsinki Declaration. Written informed consent was obtained from the parents or legal guardians of the patients prior to sample collection following a detailed explanation of schedules and contents of the study.

## Results

### Clinical features of HRV-infected children

The demographic, clinical and laboratory characteristics of all enrolled patients are shown in Table 1. HRV was detected in 98 (16.3%) of 601 NPAs. Among the HRV-positive samples, 38 (38.8%) were co-detected with other respiratory viruses. HRV-infected children were significantly younger and had a shorter fever duration than the HRV-negative group. In contrast, the frequency of chest retraction and wheezing were significantly higher in the HRV-positive group than in the HRV-negative group. The white blood cell (WBC), hemoglobin (Hb) and platelet (PLT) counts were within normal range in both groups, although there were significant differences between groups. There were no other significant differences between the laboratory results of HRV-positive and -negative patients. Between the HRV mono- and co-infection groups, there were no significant differences in clinical or laboratory data.

**Table 1:** Demographic, clinical and laboratory characteristics of HRV-positive, -negative, single-, and co-infected patients.

Clinical data	HRV (+), n = 98	HRV (-), n = 503	*P* value	HRV single infection, n = 59	HRV co-infection, n = 38	*P* value
**Age (months)**	24.7±25.6	36.9±34.3	<0.001	25.3±29.3	23.3±19.1	NS*
**Male sex, n (%)**	64 (65)	288 (57)	NS	37 (63)	26 (68)	NS
**Fever (days)**	4.0±3.1	4.9±3.6	0.018	3.9±3.0	4.3±3.2	NS
**Cough (days)**	6.7±9.1	6.0±6.5	NS	5.8±5.5	8.1±13.0	NS
**Rhinorrhea (days)**	6.2±9.4	5.2±6.7	NS	5.3±5.8	7.7±13.1	NS
**Chest retraction, n (%)**	12 (12)	22 (4)	0.002	8 (14)	4 (11)	NS
**Wheezing, n (%)**	36 (37)	128 (25)	0.022	20 (34)	16 (42)	NS
**Length of hospital stay (days)**	3.6±2.7	3.4±3.5	NS	3.5±2.5	3.8±3.0	NS
**Respiratory symptom score**	8.0±3.9	7.3±3.1	NS	8.0±3.8	8.1±3.9	NS
**WBC (x 10**^**3**^ **cells/㎣)**	11.5±4.6	10.0±4.9	0.008	11.6±4.6	11.3±4.6	NS
**Hemoglobin (g/dL)**	11.9±1.1	12.2±1.0	0.006	11.9±1.1	11.8±1.1	NS
**Platelet count (x10**^**3**^ **cells/㎣)**	403.7±143.5	348.3±134.4	<0.001	386.0±144.2	433.5±140.7	NS
**Neutrophils (%)**	49.0±17.2	51.7±18.8	NS	49.3±17.3	49.1±17.3	NS
**Lymphocytes (%)**	37.9±14.9	36.3±16.9	NS	37.8±15.5	37.8±14.1	NS
**Eosinophils (%)**	2.0±1.9	1.9±2.2	NS	1.9±1.8	1.9±2.0	NS
**CRP (mg/L)**	22.9±32.2	25.1±36.5	NS	27.2±38.1	16.8±19.1	NS
**ESR (mm/hr)**	42.7±27.3	39.9±25.6	NS	43.8±29.5	41.3±24.3	NS
**Total protein (g/dL)**	6.4±0.5	6.5±0.5	NS	6.4±0.5	6.5±0.4	NS
**Albumin (g/dL)**	4.2±1.1	4.1±0.4	NS	4.1±0.4	4.3±1.6	NS
**AST (IU/L)**	35.1±19.8	37.5±20.6	NS	36.6±23.7	32.7±11.4	NS
**ALT (IU/L)**	22.9±27.6	21.2±19.7	NS	25.2±33.1	19.6±16.0	NS

Data are presented as mean±standard deviation or number of patients (percentage).

Abbreviation: NS, not significant; WBC, white blood cell; CRP, C-reactive protein; ESR, erythrocyte sedimentation rate; AST aspartate aminotransferase; ALT, alanine aminotransferase.

The median viral load in HRV-positive patients was 2.0×10^4^ (Interquartile range 4.0×10^3^–9.8×10^4^) RNA copies/ml NPA. However, there was no relationship between the HRV viral load and either clinical symptoms or laboratory data. All relevant data, inclusive of particular epidemiologic and clinical data as well as cytokine profiles, are included in a supplemental file (S1 Table).

### Comparisons across three HRV genotypes

**Fig 1:**
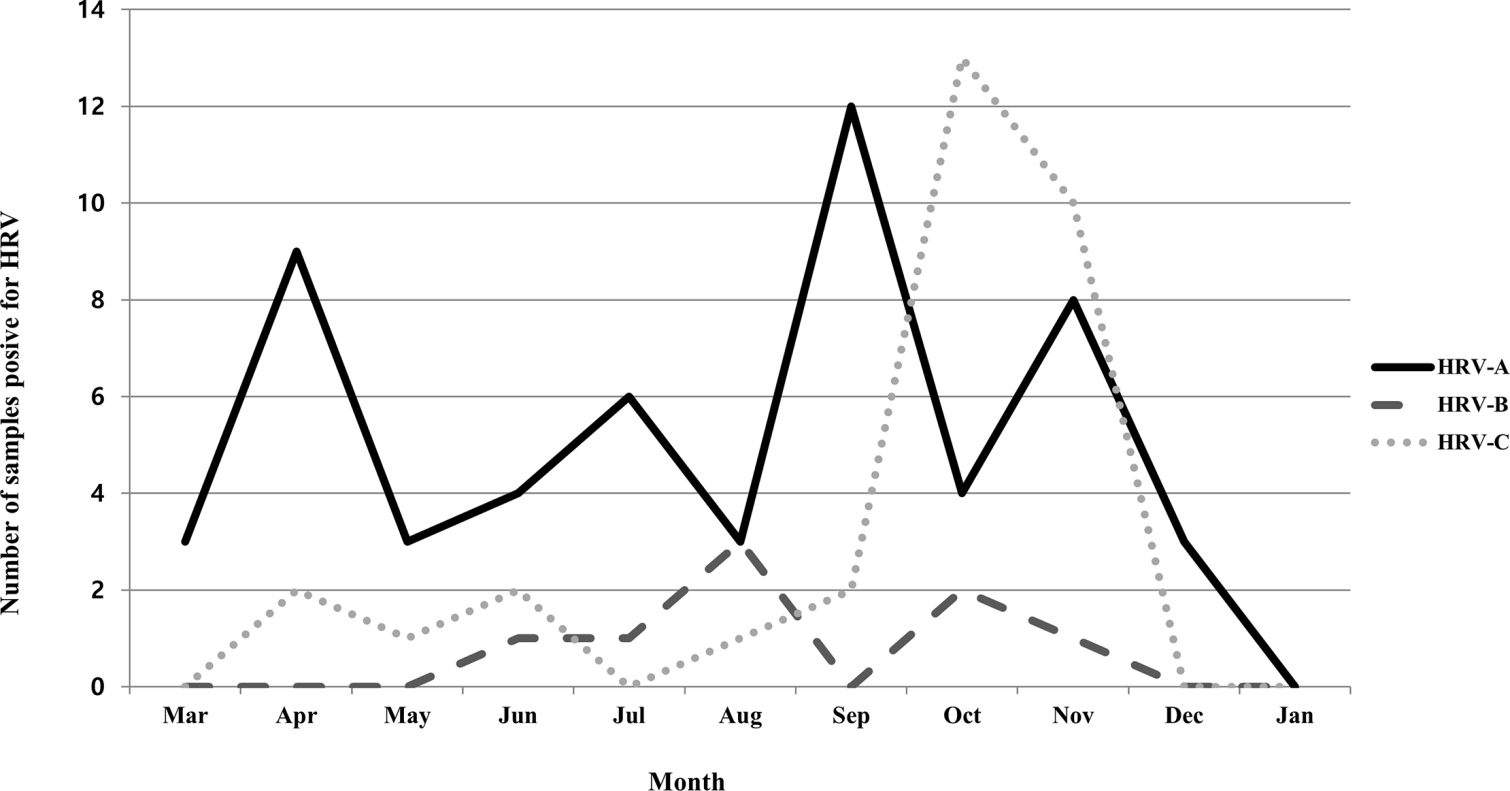
Distribution of three human rhinovirus (HRV) species by month.

The viral protein VP4/VP2 coding region was sequenced in 94 of 98 HRV-positive samples. Phylogenetic analysis demonstrated that there were 55 (58.5%), 8 (8.5%) and 31 (33.0%) cases of HRV-A, -B and -C, respectively. HRV infection occurred throughout the year during the study period, although the peak prevalence of HRV-C was observed in autumn (Fig 1). Demographic, clinical, and laboratory findings between rhinovirus genotypes are shown in Table 2. Children with HRV-B were significantly older than those infected with HRV-C. Clinical manifestations were not associated with any specific rhinovirus. The only exception was that none of the children with HRV-B infections had chest retraction, although the number of infections caused by this virus was low. There were no significant laboratory differences between HRV-A, B and C, except with regard to the WBC count, and percentage of neutrophils and lymphocytes. Regardless, these values were all within the normal range in all three groups.

**Table 2:** Demographic, clinical and laboratory characteristics of patients with HRV-A, -B and -C infections.

Clinical data	HRV-A, n = 55	HRV-B, n = 8	HRV-C, n = 31	*p* value
**Age (months)**	22.1±18.2	50.9±47.0	18.8±13.4	0.049; HRV-B vs. HRV-C
**Male sex, n (%)**	34 (62)	6 (75)	22 (71)	NS
**Fever (days)**	4.1±2.6	5.4±4.7	3.8±3.3	NS
**Cough (days)**	7.0±9.1	11.9±19.5	5.0±4.5	NS
**Rhinorrhea (days)**	6.6±9.3	11.5±19.8	4.9±4.6	NS
**Chest retraction, n (%)**	7 (13)	0 (0)	3 (10)	NS
**Wheezing, n (%)**	18 (33)	2 (25)	14 (45)	NS
**Length of hospital stay (days)**	3.5±2.5	5.0±3.9	3.6±2.7	NS
**Respiratory symptom score**	7.9±3.9	8.1±3.6	7.8±3.8	NS
**WBC (x 10**^**3**^ **cells/㎣)**	12.4±4.9	8.4±2.4	10.7±4.2	0.001; HRV-A vs. HRV-B
**Hemoglobin (g/dL)**	11.9±1.0	12.4±1.1	11.8±1.3	NS
**Platelet count (x10**^**3**^ **cells/㎣)**	421.7±141.1	343.0±104.8	392.6±157.6	NS
**Neutrophils (%)**	50.3±16.4	59.9±12.4	44.4±18.0	0.031; HRV-B vs. HRV-C
**Lymphocytes (%)**	37.6±14.8	27.6±8.6	40.9±15.2	0.021; HRV-B vs. HRV-C
**Eosinophils (%)**	1.5±1.5	1.9±1.8	2.7±2.4	NS
**CRP (mg/L)**	28.9±37.7	12.4±10.8	17.6±24.4	NS
**ESR (mm/hr)**	47.9±26.4	34.1±21.2	37.7±29.9	NS
**Total protein (g/dL)**	6.4±0.5	6.4±0.7	6.4±0.5	NS
**Albumin (g/dL)**	4.0±0.4	5.2±3.6	4.1±0.4	NS
**AST (IU/L)**	34.2±23.2	33.4±9.6	35.7±13.7	NS
**ALT (IU/L)**	21.6±31.9	17.5±5.9	26.3±23.8	NS

Data are presented as mean±standard deviation or number of patients (percentage).

Abbreviation: NS, not significant; WBC, white blood cell; CRP, C-reactive protein; ESR, erythrocyte sedimentation rate; AST aspartate aminotransferase; ALT, alanine aminotransferase.

### Nasopharyngeal aspirates cytokine analysis

The concentrations of IFN-γ, IL-4, IL-10, and TNF-α in NPA of HRV-A-, B- and C-positive patients are shown in Table 3. There were no significant differences in the NPA cytokine levels between the three groups.

**Table 3:** Cytokine levels in nasopharyngeal aspirates by HRV species.

Cytokine	HRV-A (n = 55)	HRV-B (n = 8)	HRV-C (n = 31)
**IFN-γ (pg/mL)**	2.75±5.53	2.63±±5.88	3.55±6.32
**IL-4 (pg/mL)**	0.56±2.13	Not detected^a^	0.35±0.66
**IL-10 (pg/mL)**	2.33±4.23	1.75±3.06	1.45±3.47
**TNF-α (pg/mL)**	6.05±17.36	7.13±18.55	2.74±9.91

Data are presented as mean±standard deviation or number of patients (percentage).

Abbreviation: IFN, interferon; IL, interleukin; TNF, tumor necrosis factor.

^a^below the detection limit.

## Discussion

This study demonstrates that HRV is an important cause of lower respiratory infection in young children that is associated with symptoms of respiratory distress, such as chest retraction and wheezing. Our findings corroborate several recent studies reporting that HRV, which had previously only been known to cause upper respiratory infection, could play an important role in lower respiratory infection [1,10–12]. In our study, the majority of HRV genotypes were HRV-A (58.5%) and HRV-C (33.0%). The results of this study correspond with those of earlier studies, which found that HRV-A and -C were the predominant species, although the detection order depends on the geographic region [1,13–18].

Previous studies have reported conflicting results with regard to disease severity according to HRV species. Most of the earlier studies reported that HRV-C was associated with more severe respiratory disease in children [5,13,16,18,19]. Others found no clinical differences between the three HRV species [20,21]. Further studies showed that both HRV-A and HRV-C were associated with acute respiratory infection hospital admissions, serious disease outcomes or wheezing episodes [13,15,22,23]. In our study, there were no significant differences in the clinical features, laboratory data or NPA cytokine levels between HRV-A and -C infections.

HRV-B seems somewhat different from HRV-A and -C. In accordance with previous studies [13,23–25], we found that HRV-B was the least frequently detected HRV species, and chest retraction infections caused by this virus was not observed. There are two hypotheses to explain this. It is possible that HRV-B causes a mild form of disease that does not warrant hospitalization, accounting for its lower detection rate in hospital-based studies compared to those of the other HRV species. Another possibility is that the genetic and biological fitness of HRV-B leads to its low frequency. One recent article reported that, even in healthy young children, HRV-B was the least frequently detected species of the three [26]. This finding supports the hypothesis that the frequency and virulence of HRV-B may be related to its genetic fitness, rather than selection bias.

There have been discrepant results regarding the correlation between the NPA HRV viral load and disease severity. Most previous studies reported that a high HRV viral load was correlated to clinical severity or wheezing [27–29]. However, other reports show no correlation between the viral load and severity of the lower respiratory infection [30,31]. Similarly, we did not observe a correlation between the HRV viral load and clinical features or laboratory data. Our results suggest that the difference in disease severity may be more related to the host’s susceptibility to HRV than to the viral load. Viral load can vary according to the sampling process. Therefore, the exact relationship between HRV viral load and disease severity merits further investigation.

This study has several limitations. First, the study patients were limited to hospitalized children with lower respiratory tract infections at a single center. In addition, the cytokine levels detected in this study were low and the differences were overall minor.These factors may have affected our statistical results. In addition, an asymptomatic control group was not enrolled. Including a control group is often a particular challenge in pediatric populations. Despite these shortcomings, our results contribute to the understanding of HRV’s role and immunopathogenesis in lower respiratory infection.

## Conclusions

HRV is an important pathogen of the lower respiratory tract in young children. In particular, HRV-A and HRV-C are the dominant species that cause respiratory difficulties including wheezing and chest retraction. HRV-B may cause minor lower respiratory infections in relatively older children. Further studies are needed to clarify the pathogenesis of HRV, and to define the specific clinical roles of each species.

## Supporting information

S1 Table All relevant data, inclusive of particular epidemiologic and clinical data as well as cytokine profiles in HRV-infected children. (XLSX)
